# Uncontrolled Outdoor Access for Cats: An Assessment of Risks and Benefits

**DOI:** 10.3390/ani10020258

**Published:** 2020-02-06

**Authors:** Sarah M.L. Tan, Anastasia C. Stellato, Lee Niel

**Affiliations:** 1 Faculty of Land and Food Systems, The University of British Columbia, Vancouver, BC V5H 3Z7, Canada; 2 Department of Population Medicine, Ontario Veterinary College, University of Guelph, Guelph, ON N1G 2W1, Canada

**Keywords:** cats, outdoor, disease, parasites, predation, injury, enrichment, behavior, welfare

## Abstract

**Simple Summary:**

Outdoor access for companion cats is a controversial topic. Some have suggested that outdoor access can negatively impact the welfare of companion cats through increased risks of disease and parasites, injury or death due to traffic, predation or ingestion of toxins, and becoming lost. In addition, cats can negatively influence their environments due to the predation of small birds and mammals, and they are sometimes a nuisance to human neighbors. Despite these concerns, recent estimates suggest that many owners still allow their cats outside, likely because it also provides cats with exercise, and allows them to perform natural behaviors, such as hunting, exploring, and climbing. While some suggest that cats need outdoor access, others recommend ways for cats to meet these needs indoors by providing enrichment and properly supervising cats during outdoor access. This review examines the risks and benefits associated with outdoor access for cats, as well as what is currently known about peoples’ practices, knowledge, and attitudes about the provision of outdoor access for cats.

**Abstract:**

Uncontrolled outdoor access is associated with a number of welfare concerns for companion cats, including increased risks of disease and parasites, injury or death due to traffic, predation or ingestion of toxic substances, and getting permanently separated from their owner. In addition, cats pose a threat to local wildlife due to predatory behaviors, and can sometimes be a nuisance to human neighbors. Despite these concerns, recent estimates suggest that many owners are still providing their cats with uncontrolled outdoor access, likely because it also offers welfare benefits by allowing cats to perform natural behaviors, such as hunting, exploring, and climbing. While some have suggested that outdoor access is necessary to meet cats’ behavioral needs and to prevent related behavioral problems, others have recommended various environmental enrichment strategies that can be developed to meet these needs within an indoor environment or through supervised and controlled outdoor access. This review examines the welfare issues and benefits associated with outdoor access for cats, as well as what is currently known about peoples’ practices, knowledge, and attitudes about the provision of outdoor access for cats.

## 1. Introduction

Cats are popular companion animals, with estimates of over 100 million companion cats living in homes in Canada and the United States [1,2]. In recent years, many Canadian and American humane organizations have been discouraging cat owners from allowing free-roaming outdoor access to their cats. The rationale for this recommendation is that uncontrolled (free roaming and unsupervised) outdoor access can negatively impact cat welfare by increasing susceptibility to disease from other animals, increasing the risk of vehicle collisions that may lead to death or injury, and increasing the risk of predation by other animals [3]. Additionally, many have raised concerns about cats heavily impacting their surrounding environments through predation (e.g., [4,5]) and others have suggested that cats cause disturbance to human neighbors (e.g., vocalizing, defecating in gardens) [6].

Although there are many potential welfare risks associated with uncontrolled outdoor access for cats, it has also been suggested that access to the outdoors has various benefits to cat welfare [7]. For example, it has been suggested that outdoor environments have increased natural enrichment and space for exploration, which provides for greater mental stimulation [8], and conversely, that keeping cats indoors prevents them from performing natural behaviors, such as exploring, climbing, and hunting, which might lead to boredom, frustration and increased behavioral problems [9]. Some also argue that outdoor access can improve cat health through increased physical activity [10], as they have more opportunities to roam, jump, and run.

Recent estimates suggest that owner practices surrounding the provision of outdoor access for cats vary geographically, with owners in Canada and the United States being less likely to allow their cats outdoors than owners in Europe and New Zealand. For example, one study from Canada found that 28% of owned cats are provided with uncontrolled outdoor access [1], whereas another study from New Zealand found that 95% of owners allow their cats outdoors [11]. While there has been much speculation about what might be influencing cat owner decisions about unsupervised outdoor access, there is limited research on this topic. This review explores the available literature to examine what is currently known about the risks associated with outdoor access, including potential impacts on cat health, predation of wildlife, and disturbances to non-owners, as well as the benefits of outdoor access, such as allowing for natural behaviors, preventing behavioral issues, and improving physical health. Finally, we will examine what is currently known about peoples’ practices, knowledge, and attitudes about the provision of outdoor access for cats.

## 2. Risks Associated with Outdoor Access

### 2.1. Increased Disease and Parasite Susceptibility 

While outdoors, cats are likely to interact with a variety of other companion animals and wildlife, and these types of interactions have the potential to increase the risk of disease exposure and transmission, especially if the cat is not vaccinated. One study that monitored suburban cats while outdoors using animal-borne cameras found that out of the 55 cats monitored, 28 interacted with unfamiliar cats and one interacted with wildlife during the observation period [12]. Examples of relevant communicable diseases that might be transmitted include feline leukemia virus (FeLV), feline immunodeficiency virus (FIV), bartonellosis, and rabies. Both FeLV and FIV are immunosuppressive diseases that leave cats susceptible to secondary infections, which may impair welfare and reduce their lifespan [13]. Both FeLV and FIV can be transmitted through bite wounds, and FeLV can also be transmitted through more casual contact, such as mutual grooming. In Canada and the United States, a recent study found that the seroprevalence was 3.1% for FeLV antigen and 3.6% for anti-FIV antibody in cats tested in veterinary clinics and animal shelters, and a key risk factor associated with testing positive in this study was outdoor access [14]. One study from the United States found that 73% of outdoor cats were known to be vaccinated against FeLV [15]. However, vaccination does not completely protect against infection, with one recent field study suggesting a protective rate of only 56% for the FIV vaccine Fel-O-Vax FIV^®^ [16]. Similarly, there are increased risks of acquiring parasites while outdoors [17]; these can include internal parasites, such as helminths and protozoans, and external parasites, such as fleas and ticks. Internal parasite infections can impair welfare through general malaise, and can also lead to mortality, particularly in very young and old animals [18]. External parasite infestation can directly impair welfare through discomfort associated with bites and related irritation. One study in the UK found that 21% of cats visiting veterinary clinics had fleas present during inspection, and that 8% of cats showed signs of flea allergy dermatitis [19]. Interestingly, almost half of the owners of cats with fleas did not know that their cat was affected. In some cases, external parasites can introduce other more serious diseases, such as Lyme disease, but, with limited research on this topic, the impacts on cat welfare are unclear [20].

The risk of contracting some diseases and parasites varies regionally and seasonally [21,22]. Sanguinetti-Morelli and colleagues [22] found cat-scratch disease varies seasonally in France, with the majority of cases occurring between September to December and peaking in November, which is comparable to the United States and Japan. In Canada, the prevalence of individual parasites, such as taeniid and capillarid eggs, statistically differs between the East and Pacific regions [21]. Similarly, rabies was found to be greater than 10 times more prevalent and affected more mammal species in Ontario, Canada than in British Columbia, Canada [23]. Therefore, cats are likely more susceptible to contracting a disease or parasite that could kill or cause temporary or permanent injuries in some regions than others. 

### 2.2. Increased Risk of Trauma, Predation, and Poisoning

Road traffic accidents have also been discussed as a major concern for cats with uncontrolled outdoor access. Loyd and colleagues [12] examined cat risk-taking behaviors using an animal-borne camera with cats in a suburban area in the United States, and found that 45% of cats crossed a road during the monitoring period. One study examined causes of death in cats brought into a Canadian veterinary clinic, and found that trauma was the cause of 39% of sudden deaths in cats, with 87% of those cases due to motor vehicle accidents [24]. Trauma was characterized by the presence of damage, such as lesions, rupture or displacement of internal organs, hemorrhaging, and broken bones, which could cause major discomfort, pain, and long-term consequences (e.g., leg amputations) or death. Similarly, road traffic accidents were cited as a common reason for cat deaths in the United Kingdom. In exploring the impact of road traffic accidents, 16 out of 127 cats involved in a motor accident arrived at the hospital already dead, 11 died subsequently to the visit, and 25 developed severe injuries resulting in long-term health issues, such as limb amputations, ongoing bladder problems, and ruptured diaphragms [25]. Thus, allowing cats uncontrolled outdoor access increases the risk of cat fatalities from collisions. Additionally, collisions can inflict extensive damage to organs or extremities, which can develop into acute or chronic long-term health concerns, negatively impacting cat welfare.

Wildlife and dog predation on cats can also inflict significant trauma and result in death. Olsen and Allen [24] reported 3 of 31 cat trauma fatalities were due to suspected dog bites, although it is unclear whether these bites occurred within the home or while free roaming. Besides dog predation, coyote attacks appear to be another common concern for cats that freely roam outdoors. Analyses of urban coyote scats have found that both cat and dog residues are present, ranging from 1.2% domestic dog and cat residues in urban coyote scat in Calgary [26], to 13.6% residue of domestic cat in Los Angeles [27]. It has been suggested that coyotes rely on pets, including cats, as a food source, especially during the winter and spring when their regular prey (e.g., birds and rabbits) may be inactive or hibernating [28]. However, it is unclear whether cats were scavenged or preyed upon, which would influence the level of risk coyotes pose to outdoor cats. Lastly, traumas can also arise from agonistic interactions with other cats in response to overlapping territories and the sharing of resources [29].

While limited research has been done to explore the impact of toxic hazards on cat morbidity and mortality, there are various poisonous substances that cats can potentially be exposed to while outdoors. Loyd and colleagues [12] found that 20 out of 55 cats (36%) in a suburban area ingested liquids and solids that were not provided by the owner during unsupervised outdoor access. The potential for accidental poisoning (e.g., drinking from a contaminated puddle) from pesticides’ or insecticides’ runoff would likely be of concern in rural or farm regions. Additionally, since cats often hunt rodents [30], which are a pest control target of rodenticides, they could be affected through secondary poisoning following consumption of a contaminated carcass. Rodenticides are relatively slow acting, and the effects of related poisoning, including internal bleeding, vomiting, and anorexia, would be expected to last for a few hours to multiple days, possibly resulting in death [31]. A study in New Zealand observed that secondary poisoning a of rabbit-controlling chemical, sodium monofluoroacetate, contributed to a decline in rabbit predators [32], suggesting it is reasonable to consider secondary poisoning as a risk for cats that hunt. However, no studies that we are aware of have specifically examined secondary poisoning of cats. Chemicals, such as ethylene glycol in antifreeze, are also a potential concern for accidental intoxication. A study conducted at the Colorado State University Veterinary Teaching Hospital recorded 104 cases of cat intoxication between 1979 and 1986, where approximately 29% of cases were due to ethylene glycol, resulting in death for 43% of those cases [33]. However, the overall the number of reported cases of ethylene glycol intoxication during this period was relatively low. Lastly, a diverse population of plants are also toxic to cats and are commonly found in open access areas, like gardens, meadows, and forests. For instance, cats are highly sensitive to lilies, where just two leaves or a part of the flower (e.g., stamen, pollen) can cause renal damage, salivation, vomiting, or death [34]. However, knowledge of the exact toxins and toxicity levels causing these effects is unknown. Given the variety of substances that are found outdoors that are potentially toxic to cats, there is a need for more research to explore how toxicity exposure varies between regions, and how risk of intoxication in cats is impacted by outdoor access.

The risk of predation, injury, or death in outdoor cats might vary depending on whether cats reside within a rural or urban environment. One study indicates that cats have larger home ranges when housing densities are low, such as in rural environments [35]. It is possible that outdoor cats that roam further from their residence are at an increased risk of predation due to an increased potential for contact with predators. While there is no evidence to support this specific hypothesis, it is likely that more wild predators are present close to rural households in comparison to urban households. Anthropogenic risks are also likely to differ between urban and rural environments. One potential assumption is that most anthropogenic risks, such as traffic accidents and poisoning, will be higher in urban areas due to increased density, but at least one study suggests that these assumptions might be incorrect; a recent study identifying risk factors for road traffic accidents involving cats within the United Kingdom found that rural locations were associated with a higher odds of accidents in comparison to towns, cities, or suburban locations [36]. The authors suggest that accidents are reduced in urban areas because these areas have more speed restrictions and improved visibility for drivers, and consistently heavy traffic might allow cats to learn to avoid vehicles. Thus, further research is needed to explore which types of environments are associated with increased risks to outdoor cat welfare.

### 2.3. Contribution to Homeless and Feral Cat Populations

Cats with outdoor access are also at a higher risk of getting lost and contributing to homeless and feral cat populations. In 2016, 56% of the cats received into Canadian shelters were strays [1]; based on a total intake of 114,131 cats, it is estimated that 63,456 were strays. While 68% of stray dogs were returned to their owners, only 10% of stray cats were returned. The Canadian Federation of Humane Societies report suggested that this difference may have been a result of owner abandonment, or the belief that cats are more disposable than dogs. In the United States, it is estimated that there are 30 to 40 million stray and feral cats [2]. Similar to Canadian intakes, 880,415 of 1,648,089 cats (53.4%) received into American shelters were strays [37]. Compared to Canadian shelters, American shelters had a lower rate of return for both dogs and cats, but dogs (37%) still had a higher return rate than cats (5%). As cat overpopulation remains a challenge in Canada and the United States, managing the risk of abandonment or lost cats is an important aspect to minimize the number of stray cats.

### 2.4. Negative Impacts on Wildlife Welfare

Uncontrolled outdoor cats can also have negative impacts on other species, including wildlife. For instance, cats have caused local extinctions of species, exterminated species endemic to islands, and have become an issue to a variety of ecosystems [38,39,40]. Domestic cats are obligate carnivores and retain highly developed predatory skills even when kept as companion animals [41]. Various studies have assessed predation by companion cats using owner reports, and a summary provided by Loyd and colleagues [42] suggests that the primary predation targets are small mammals, followed by birds. Using animal-borne video cameras, one study conducted in a suburban area on the southeastern United States found that 44% of companion cats with free-roaming outdoor access engaged in predation of wildlife while outdoors [42]. Predation targets in this particular study were specific to the region, and included reptiles, mammals, and invertebrates, and predation rates varied with the season. Most of the cats that were successful at hunting captured one or two prey items during the 7-day observation period, but some cats captured up to five items. Interestingly, only 23% of prey items were brought back to the household, suggesting that owner reports of cat hunting behavior likely underrepresent total predation rates.

It can be challenging to estimate the contribution of companion cats to wildlife predation since most studies provide overall estimates that include homeless and feral populations. A recent review estimated that cats cause between 6.3 and 22.3 billion mammal mortalities and between 1.3 and 4 billion bird mortalities annually in the United States [43]. In Canada, it is estimated that cats kill between 100 and 350 million birds annually [44]. As stated above, the majority of the fatalities were attributed to feral cats so the impact of companion cats with outdoor access is unknown. However, feral cat populations within Canada and the United States arise through mismanagement of companion populations; therefore, the overall numbers represent the total impact of outdoor cat access. Considering a cat’s ability to efficiently hunt and kill, predation can have serious repercussions on wildlife populations, particularly in isolated communities (e.g., New Zealand, Australia, and Hawaii), where native wildlife has evolved without predators like cats, and consequently has limited defense mechanisms [45]. In a recent survey, the public in New Zealand expressed concern about cat predation of native wildlife, with the highest level of concern related to predation by feral cats, followed by unmanaged stray cats, managed stray cats, and finally, owned cats [5]. In addition, the majority of participants suggested that cats should not be owned near endangered or protected species. While feral cats and cat colonies are the main concern, predation from domestic cats and the introduction of cats into isolated areas can exacerbate threats to vulnerable inhabitant species.

A number of solutions have been suggested for reducing cat predation success, including specialized high visibility collars that also interfere with prey capture (e.g., CatBibs) and collar-mounted devices that warn of approach (e.g., bells). In a recent study, many New Zealand cat owners reported that they use belled collars for identification as well as to reduce their cat’s hunting behavior [46]. In terms of efficacy at preventing predation, both bells and CatBibs have been found to reduce the amount of prey caught by pet cats [47,48]. 

Cats also have the potential to negatively impact wild carnivore populations. Some authors have suggested that cats might compete with wild carnivores for food sources [49], although evidence to support this is lacking. Domestic cats have also been found to interbreed with wildcat populations in some regions. For example, hybridization between European wildcats and domestic cats has been detected and raised as an issue in Hungary [50], France [51], and Portugal [52]. This hybridization might be a threat to these wildcat populations, and also has the potential to affect domestic cat welfare if policies are put in place to protect the genetic integrity of wildcats through trapping or culling.

### 2.5. Negative Impacts on Humans and Relevant Municipal By-Laws

Cats are known to carry a variety of diseases that are considered zoonotic, and are thus transmittable to humans. This transmission can occur through vectors, such as fleas and ticks, that cats bring into the household, or through physical contact, bites and scratches, airborne passage of pathogens, or contact with feces in soil or water [53]. Most concern with disease transmission is in relation to toxoplasmosis gondii [54], as felids are the definitive hosts for the sexual stage of this protozoa and are the only hosts that accommodate both the asexual and sexual stages, and shed the environmentally resistant oocysts in their feces [55]. Risk of transmitting diseases to humans is greater for outdoor cats, as indoor cats are less likely to be exposed to disease risks due to their reduced interaction with other wild animals and the environment [53].

Outdoor cats can also be a nuisance to human neighbors through digging and defecation on private property, predation on birds at birdfeeders, and excessive nocturnal vocalizations, particularly associated with mating and fighting [5,6,56]. Many municipalities in Canada and the United States have by-laws that address these types of issues related to outdoor cats, and even within a relatively close geographical area, regulations can vary significantly. For example, in southern Ontario in Canada, some municipalities address cat licensing but do not provide specific regulations restricting roaming cats (e.g., Guelph, ON [57]), whereas others have specific types of restrictions, such as allowing roaming cats but prohibiting cats from damaging a neighbor’s property (e.g., Toronto, ON [58]), or completely banning owned cats from roaming outside the owner’s property (Oakville, ON [59]). In contrast, more extreme restrictions on cat ownership and movement are found in other countries, such as Australia and New Zealand. For example, some local councils in Australia have implemented confinement and no-cat zones [6], and in New Zealand some residential areas are cat free [60].

## 3. Welfare Benefits Associated with Outdoor Access

### 3.1. Natural Behaviors

Outdoor access allows cats to freely interact with a dynamic environment, promoting natural behaviors, such as hunting, exploring, and climbing. While it has been suggested that animal welfare can be impaired through restriction of natural behaviors [61], no research that we are aware of has actually assessed the effect of outdoor access on cat welfare. This is a complex topic that would require assessment of the provision in naïve animals with no prior access, as well as restriction in animals who had previously been provided with access, since responses might differ in these populations. Furthermore, research would be required to determine whether particular indoor enrichments are sufficient for stimulating and replacing these behaviors in the indoor environment. Here, we briefly review some of the natural behaviors that have been suggested to be important for promoting good welfare in cats, and suggest potential enrichments that might be appropriate for stimulating these behaviors in an indoor environment.

Domestic cats retain strong instincts to perform predatory behaviors, such as stalking and hunting, even with indoor housing and sufficient food available [41]. As noted above, Loyd and colleagues [42] found that 45% of cats engaged in predatory behavior when outdoors during their 7-day observation period. However, some have suggested that it is not ethical to encourage cat predation of wildlife through outdoor access [4]. As an alternative, enrichment tools that simulate feeding and hunting opportunities can be provided to replicate hunting behaviors in indoor environments. Hiding food items, scattering feed, and using puzzle feeders can mimic investigatory and consummatory aspects of the hunting process, as well as provide mental stimulation through problem solving [62]. Wands with feathers or fur can imitate flying or ground prey when lifted into the air or shaken on the floor, where a catch of the wand or toy can mimic a successful capture [63], stimulating natural behaviors, such as stalking, pouncing, jumping, and biting. While provision of these types of enrichment is likely to improve the welfare of indoor cats, there is also the potential that it might stimulate and train hunting behavior in cats that are also allowed uncontrolled outdoor access. 

Kasbaoui and colleagues [8] found that on average, outdoor owned cats in England travelled 4.4 kilometers a day, suggesting cats spend a significant portion of their day travelling and exploring. Exploration allows cats to experience variety in a range of different environments, and also provides physical exercise, which can be good for cat health. While it is difficult to provide the quality and quantity of enrichment and space available in outdoor environments within indoor spaces, simple methods to promote exploration in indoor environments include introducing variability and unpredictability into the home environment [63]. For instance, spatial home diversity can be enhanced by providing shelves to increase vertical space, and by introducing novel objects, such as boxes, cat tunnels, or paper bags, and alternating their locations.

Lastly, climbing, perching, and monitoring of the surrounding area are important aspects of cat behavior that can be limited by indoor housing. Cats are predatory animals, and it has been suggested that they prefer to be above level, using climbing for concealment and observing their surroundings and prey [64]. Elevated structures, such as shelves, climbing posts, cat condos, and windowsills, can be implemented to supply cats with these vantage points. Additionally, elevated structures and platforms increase the amount of territory area available to hide and explore, improving overall housing quality. Ajar or screened windows can also provide cats with the ability to hear, smell, and monitor their outdoor environments [63] without risking the various associated hazards.

While the provision of opportunities for the natural behaviors listed above has been suggested to be important for cat welfare, a recent systematic review examined the impact of environmental and social enrichments on cat welfare and found that there is limited research to support the efficacy of most recommendations [65]. The authors concluded that further high-quality research is needed on this topic, and in particular, studies are needed to explore the impact of complex environments involving multiple types of enrichments that might interact with each other.

### 3.2. Prevention of Behavioral Problems

As discussed, outdoor access allows cats to demonstrate natural behaviors, and some have suggested that if these behaviors are prevented, it might lead to frustration, boredom, and the development of problematic behaviors, such as aggression, inappropriate urine marking or toileting, and furniture scratching [66]. Behavioral issues often indicate that welfare is negatively impacted, and the presence of these behavioral issues also puts cats at increased risk for other welfare issues like relinquishment or abandonment [67]. While negative impacts of restricted outdoor access have been suggested as possible risk factors for various behavioral issues, there has been limited empirical research to examine whether limitations on outdoor access actually lead to behavioral problems. For example, one study found that owners identified limitations on outdoor access as a potential cause of urine marking in cats, but this was not empirically tested [68]. Other studies have examined behavioral issues in cats with and without outdoor access. One study found that higher levels of house soiling and scratching of household objects were reported for indoor cats [69], and another found that cats with behavior problems, such as aggression and inappropriate elimination, were less likely to be allowed outdoors than cats from a control group without behavior problems [9]. However, it is important to consider whether cats had experience with outdoor environments prior to being restricted indoors, since increased frustration following behavioral restriction in these cats might be more likely to lead to behavioral issues.

For indoor-only cats, providing environmental enrichment to improve housing qualities (e.g., spatial, behavioral) might help minimize boredom, and unwanted behaviors. Strickler and Shull [70] explored whether the presence and absence of different toys and activities influenced behavioral problems in indoor cats. For example, cats that were not provided with string toys were more likely to exhibit inter-household aggression, aggression towards visitors, and aggression towards outdoor cats. An increase in behavioral issues was also found in cats that were not provided with access to the following toys and activities: tricks (cognitive/social), ball without bell (play/hunting), fishing pole (play/hunting), string (play), massage (social), feather (play/sensory), and animal companion (social). These findings suggest that providing enrichment opportunities that engage the cat in play, cognitive, social, and hunting behaviors can reduce unwanted behaviors, such as inappropriate urination and aggression towards owners. 

### 3.3. Exercise

Confinement to indoor environments has been suggested to contribute to obesity in cats, likely due to reduced opportunities for exercise. One study found that one-year-old cats without outdoor access had a greater risk of being overweight or obese [10]. In companion cats in developed countries, obesity is considered a significant welfare concern. Overweight and obese cats are at a higher risk of developing various diseases that can impact cat welfare, such as urogenital disorders, cardiorespiratory diseases, and metabolic problems [10]. The most recent estimates for cats in the United States reported that 28.7% of cats were overweight and 2.2% were obese [71]. Recent estimates from New Zealand suggest that cat weight is still a significant issue, with 21.9% of cats being overweight and 2.6% being obese [72]. However, given that cats are more likely to be allowed outside in New Zealand, further research is needed to determine whether indoor restriction is actually a risk factor for weight issues in cats, and if so, whether particular types of enrichment can be used to reduce obesity. While it is impossible to provide as much space as is available to cats when free roaming outdoors, owners can increase exercise in their indoor environment with a diversity of enrichment and toys (e.g., feather rods, running wheels) and with frequent social interactions. 

### 3.4. Alternative Outdoor Access

There are obvious welfare benefits associated with outdoor access for cats; however, controlled outdoor access can be provided in ways that encourage important natural behaviors while minimizing the risks that occur when cats are left unsupervised. Options for controlled outdoor access include access to enclosed outdoor environments, such as secure enclosed outdoor spaces (e.g., catios) or yards made secure with cat-specific fencing, leash or harness walks, and tie-outs, where cats are connected to a secure long line. While controlled outdoor access imposes some limits on natural behavior, with fewer hunting opportunities than uncontrolled access, the extra space and stimulation still increases the potential for cats to explore and interact with their environment. Additionally, cats on leashes or harnesses require positive training to allow a safe and calm environment for the cat to choose where to walk [63]. Overall, even if cats are leashed or fenced in, outdoor access can provide auditory, olfactory, and visual enrichment through environmental stimulants (e.g., birds chirping and flying) and are available options in which cats can interact with their natural environment. However, these forms of outdoor access might result in frustration due to perceived restriction, particularly if cats previously had uncontrolled outdoor access; therefore, research is needed to explore the impact of these alternatives on welfare and the development of behavior problems.

## 4. Human Attitudes about Provision of Outdoor Access for Cats

Many people believe that cats are more independent than other companion animals, such as dogs [73], which leads to perceptions that cats are capable of independent living, with limited need for intervention or care from owners. While free-roaming dogs are relatively rare in most highly populated regions of Canada and the United States, free-roaming cats are reported to be relatively common [2,74]. It is possible that owner attitudes about cat independence and self-sufficiency increase the likelihood of allowing uncontrolled outdoor access, but this topic requires further study.

As detailed above, one of the most significant concerns associated with outdoor cats is the potential threat to wildlife due to predation. There has been limited research on this topic, but one study suggests that owner attitudes about cat hunting behavior are somewhat inconsistent. The authors found that the most prominent viewpoint was that participants did not like their cat’s hunting behavior but tolerated it because it was perceived as “natural” [75]. However, if owners were able to recognize their cat as a hunter, they were more willing to take action to mitigate the behavior, and many owners also accepted some responsibility for their cats’ potential to cause nuisance to others’ pets or property. Given these perspectives, cat owners need to recognize the impact their cat has on other animals and environments before they will be open to taking action to prevent these impacts. Moreover, a survey of Florida and Hawaii stakeholders (general public, conservation community, and animal-welfare community) revealed that public tolerance for outdoor cats depended on the type of wildlife at risk, as Hawaiian stakeholders were more concerned about outdoor cats than those from Florida [76].

While many humane societies educate owners regarding the risks associated with uncontrolled outdoor access for cats (e.g., [3]), a large proportion of owners continue to allow cats to roam freely. Recent estimates from Canada suggest that 28% of owners allow cats to free roam without supervision [1], which suggests that there is either a lack of awareness amongst some owners about the specific risks of outdoor access, or that these owners believe that the benefits outweigh the risks.

To date, only a few studies have examined cat owner practices and attitudes about the provision of outdoor access for companion cats. A study from Victoria, Australia surveyed cat owners on their attitudes about cat containment and found that 41% of owners contained their cat to their property, and that the majority of cat owners believed containment was important to protect cats from injury and to protect wildlife from predation [6]. However, other research indicates that many owners are less willing to confine their cats to protect wildlife (e.g., [77,78,79]). A survey from New Zealand, targeted to veterinarians and cat owners to prioritize cat management behaviors, revealed that bringing cats in at night was likely to be adopted by cat owners, but confining cats inside 24-h per day was not supported by veterinarians and was unlikely to be adopted by cat owners [80]. Clancy and colleagues [15] surveyed 184 cat owners in Massachusetts in the United States on which factors influence owner provision of outdoor access for cats. Owners reported that 40% of cats had some degree of outdoor access, although the authors did not differentiate between controlled (e.g., in a controlled area or under direct supervision) and uncontrolled access. Overall, cats acquired from shelters were significantly less likely to have outdoor access than cats acquired as strays, which they hypothesized was due to increased educational efforts from humane societies. No other owner demographic variables or cat characteristics were significant, but the questionnaire was relatively limited in scope and did not ask about owner attitudes. Thus, additional research involving a broader population and more detailed questioning on owner attitudes of outdoor access, cat characteristics, and cat management related to outdoor access is needed.

## 5. Conclusions

Housing that encourages natural motivated behaviors is important for good welfare in cats because it encourages physical activity and prevents negative affective states, such as boredom and frustration. Uncontrolled outdoor access allows these natural behaviors, but it also poses a number of significant health and welfare risks, and the overall benefits of the performance of these behaviors is unknown. While there are potential welfare impairments with indoor restrictions, a number of enrichment strategies have been suggested for counteracting these effects to promote the health, affective states, and performance of natural behaviors of indoor cats. Further research is needed to assess the efficacy of these strategies and to determine whether the welfare of cats is impaired by restriction. To develop effective educational strategies aimed at reducing the impact of uncontrolled outdoor access for cats, further research is necessary to improve our understanding of owner practices and attitudes towards outdoor access for cats.
